# Human and entomological surveillance of West Nile fever, dengue and chikungunya in Veneto Region, Italy, 2010-2012

**DOI:** 10.1186/1471-2334-14-60

**Published:** 2014-02-05

**Authors:** Federico Gobbi, Gioia Capelli, Andrea Angheben, Mario Giobbia, Mario Conforto, Marzia Franzetti, Anna Maria Cattelan, Enzo Raise, Pierangelo Rovere, Paolo Mulatti, Fabrizio Montarsi, Andrea Drago, Luisa Barzon, Giuseppina Napoletano, Francesca Zanella, Francesca Pozza, Francesca Russo, Paolo Rosi, Giorgio Palù, Zeno Bisoffi

**Affiliations:** 1 Centre for Tropical Diseases, Sacro Cuore-Don Calabria Hospital, Negrar, Verona, Italy; 2 Istituto Zooprofilattico Sperimentale delle Venezie, Legnaro, Padova, Italy; 3 Division of Infectious Diseases, Treviso Hospital, Treviso, Italy; 4 Division of Infectious Diseases, Vicenza Hospital, Vicenza, Italy; 5 Division of Infectious Diseases, Padova Hospital, Padova, Italy; 6 Division of Infectious Diseases, Rovigo Hospital, Treviso, Italy; 7 Division of Infectious Diseases, Venezia Hospital, Venezia, Italy; 8 Division of Infectious Diseases, Legnago Hospital, Legnago, Italy; 9 Entostudio, Brugine, Padova, Italy; 10 Department of Molecular Medicine, University of Padova, Padova, Italy; 11 Regional Reference Laboratory for Infectious Diseases, Microbiology and Virology Unit, Padova, University Hospital, Padova, Italy; 12 Department of Public Health, ULSS 20, Verona, Italy; 13 Department of Public Health and Screening, Veneto Region, Venezia, Italy; 14 Regional Centre for Emergencies of Veneto, Venezia, Italy

**Keywords:** Dengue, Chikungunya, West Nile, Surveillance

## Abstract

**Background:**

Since 2010 Veneto region (North-Eastern Italy) planned a special integrated surveillance of summer fevers to promptly identify cases of West Nile Fever (WNF), dengue (DENV) and chikungunya (CHIKV). The objectives of this study were (i) To increase the detection rate of imported CHIKV and DENV cases in travellers from endemic areas and promptly identify potential autochthonous cases.(ii) To detect autochthonous cases of WNF, besides those of West Nile Neuroinvasive Disease (WNND) that were already included in a national surveillance.

**Methods:**

Human surveillance: a traveler who had returned within the previous 15 days from endemic countries, with fever >38°C, absence of leucocytosis (leukocyte count <10,000 μL), and absence of other obvious causes of fever, after ruling out malaria, was considered a possible case of CHIKV or DENV. A possible autochthonous case of WNF was defined as a patient with fever >38°C for <7 days, no recent travel history and absence of other obvious causes of fever. Entomologic surveillance: for West Nile (WNV) it was carried out from May through November placing CDC-CO2 traps in five provinces of Veneto Region, while for DENV and CHIKV it was also performed around residences of viremic cases.

**Results:**

Human surveillance: between 2010 and 2012, 234 patients with fever after travelling were screened, of which 27 (11,5%) were found infected (24 with DENV and 3 with CHIKV). No autochthonous case of DENV or CHIKV was detected. Autochthonous patients screened for WNF were 408, and 24 (5,9%) were confirmed cases. Entomologic surveillance: the WNV was found in 10, 2 and 11 pools of *Culex pipiens* from 2010 to 2012 respectively, in sites of Rovigo, Verona, Venezia and Treviso provinces). No infected *Aedes albopictus* with DENV or CHIKV was found.

**Conclusions:**

Veneto is the only Italian region reporting WNV human cases every year since 2008. WNV is likely to cause sporadic cases and unforeseeable outbreaks for decades. Including WNF in surveillance provides additional information and possibly an early alert system. Timely detection of DENV and CHIKV should prompt vector control measures to prevent local outbreaks.

## Background

In Italy, the first outbreak of West Nile virus (WNV) infection was reported in 1998 among horses residing in Tuscany region [1]. The virus re-emerged in Italy in 2008, when equine and human cases of West Nile neuroinvasive disease (WNND) were notified in Veneto and Emilia Romagna Regions [2,3].

Moreover, veterinary and entomological surveillance documented that WNV infection was widespread in the same areas in North-Eastern Italy, with notification of 251 outbreaks in equine stables and viral isolation in resident bird species [4].

Autochthonous cases of chikungunya (CHIKV) in Europe were reported in the well known outbreak in Emilia Romagna in 2007 [5] and in France in 2010 [6]. Autochthonous cases of dengue (DENV) in Europe were reported in 2010 in France [6,7] and in Croatia [8,9] while in 2012 a large outbreak occurred in the island of Madeira (Portugal) [10].

**Figure 1:**
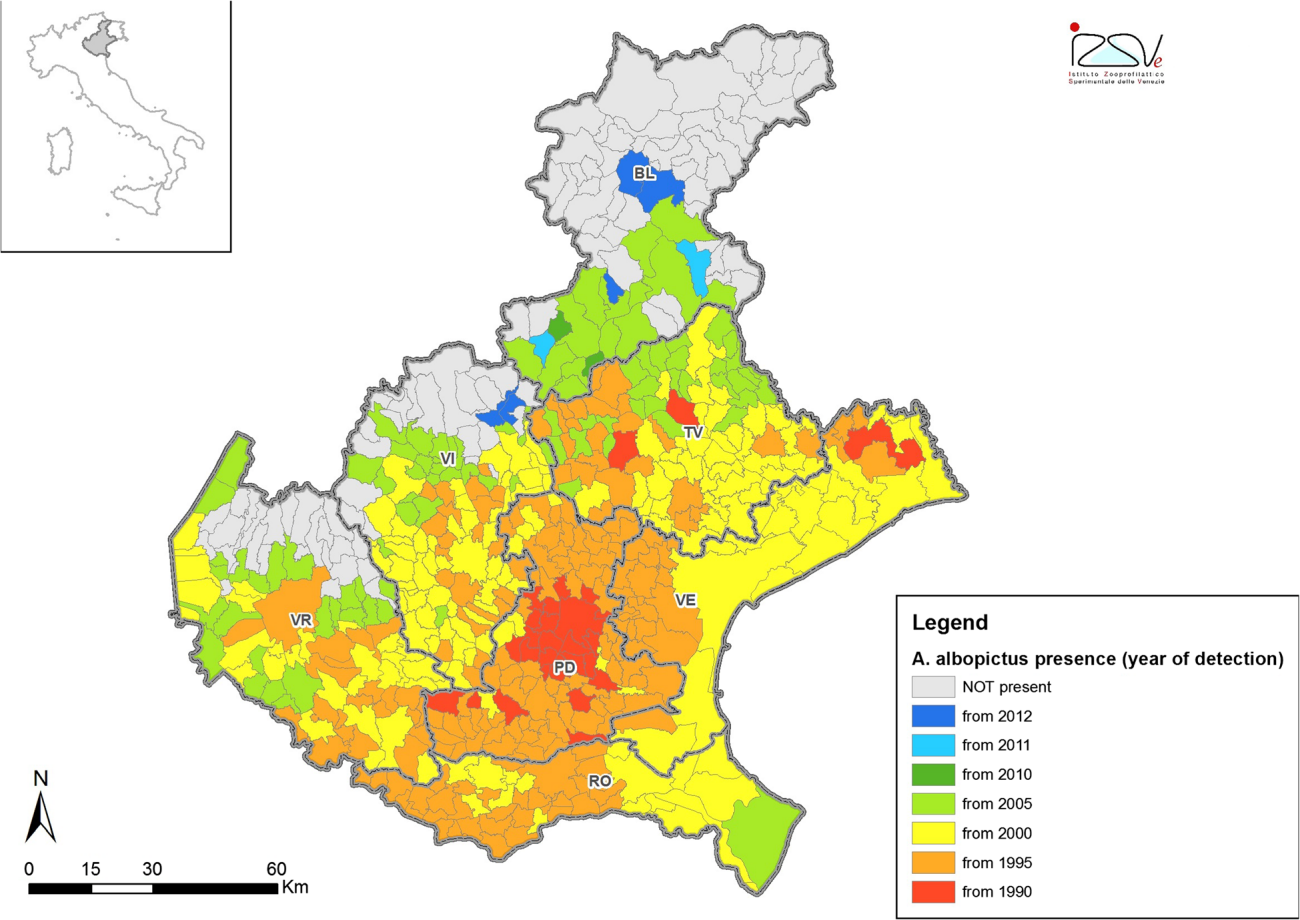
**Distribution of *Ae. albopictus *** **in Veneto region, according to the year of first detection.**

So far no autochthonous cases of DENV and CHIKV have been reported in Veneto region, but the presence of *Ae. albopictus* highlights the potential risk of outbreak following the introduction of a viremic host. This mosquito species, after the first report in Italy (Genoa, Liguria region) in 1990 [11], was then reported in Veneto in 1991 and rapidly spread to the whole territory of the region [12] (Figure 1).

In view of the appearance of human WNV in our Region on one side, and of the established and massive presence of *Ae. albopictus* on the other, since 2010 Veneto Region (North-Eastern Italy) has planned a special integrated surveillance of summer fevers to promptly identify cases of West Nile Fever (WNF), DENV and CHIKV.

Before the onset of special surveillance, in 2008 one case of CHIKV, two cases of DENV (all imported) and 1 case of WNND had been reported; four more cases of WNND and one case of West Nile Fever (WNF) had been identified retrospectively [3,13] (Table 1).

**Table 1:** Cases of the three diseases notified in Veneto Region, 2008-2012

**Year (15**^**th **^**June-31**^**st **^**October)**	**Imported dengue cases/patients screened**	**Imported chikungunya cases/patients screened**	**Autochthonous WNF cases/patients screened**	**Autochthonous WNND cases**
2010*	**14/79 (17.7%)**	**1/79 (1.2%)**	**4/38 (10.5%)**	**3**
**2011**	**3/29 (10.3%)**	**0/29 (0%)**	**3/51 (5.8%)**	**10**
**2012#**	**7/126 (5.5%)**	**2/126 (1.5%)**	**17/319 (5.3%)**	**21**
**TOT 2010-2012**	**24/234 (10.2%)**	**3/234 (1.2%)**	**24/408 (5.9%)**	**34**

*Surveillance only started the last week of July. #Surveillance was prolonged until 30^th^ of November.

In 2009 four cases of imported DENV and 6 cases of WNND (one fatal) had been reported [14], with no case of CHIKV nor of WNF.

The success of the pilot phase in 2010 [15] prompted the Regional authorities to extend the project to two more years (2011-2012), as part of the integrated surveillance of arboviral diseases, along with veterinary and entomologic surveillance.

## Methods

### Objectives

The main objectives of human surveillance were:

a) To increase the detection rate of imported CHIKV and DENV cases in travellers from endemic areas, including new immigrants and settled immigrants visiting relatives and friends (VFR), and to promptly identify potential autochthonous cases;

b) To detect autochthonous cases of WNF, along with those of WNND already included in regular surveillance, in order to obtain a more reliable picture of the disease transmission in the region.

The main objectives of entomological surveillance were:

c) To detect DENV or CHIKV in *Ae. albopictus* vectors, in case of the report of a viremic human case of DENV or CHIKV;

d) to predict the WNV circulation in the area through the search of the virus in *Culex pipiens* vectors.

### Human surveillance

**Figure 2:**
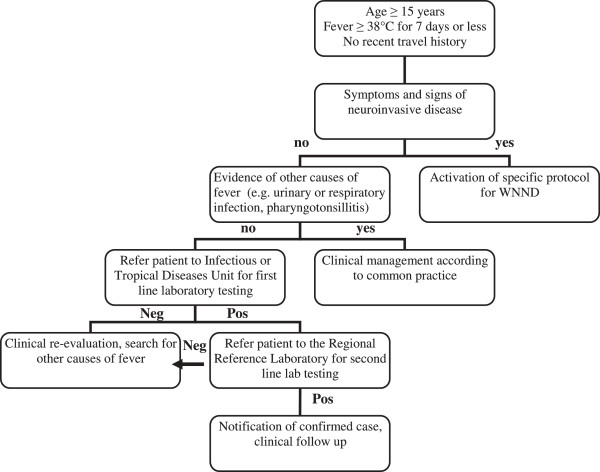
**Algorithm for detection of possible cases of West Nile Fever, Veneto Region, since 2011.** N, no; Y, Yes; WNND, West Nile neuroinvasive disease; neg, negative; pos, positive.

**Figure 3:**
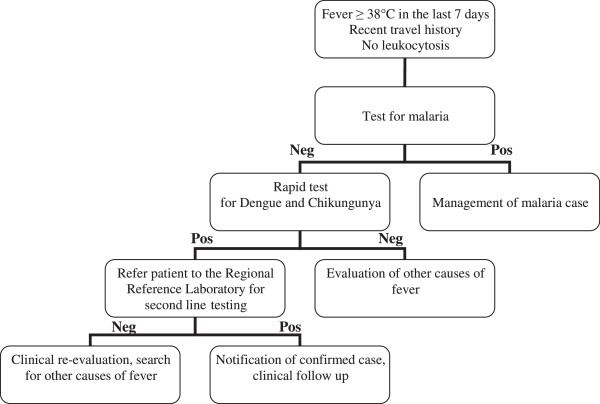
**Algorithm for management of possible cases of dengue and chikungunya, Veneto Region, since 2011.** Neg, negative; pos, positive.

Case definition are reported in Figures 2 and 3. A traveler who had returned within the previous 15 days from endemic countries for DENV or CHIKV, with fever >38°C, absence of leucocytosis (leukocyte count <10,000 μL), and absence of other obvious causes of fever, after ruling out malaria, was considered a possible case of DENV or CHIKV.

If rapid tests for DENV or CHIKV resulted positive, the patient was considered a probable case. Rapid tests included detection of anti-CHIKV IgM with the OnSite Chikungunya IgM Combo Rapid Test (CTK Biotech, Inc., San Diego, CA, USA), anti-DENV IgM and IgG with the Dengue Quick test (Cypress Diagnostics, Langdorp, Belgium) and of DENV nonstructural protein (NS) 1 antigen with the Dengue NS1 Ag STRIP (Bio-Rad Laboratories, Hercules, CA, USA) on serum samples. Confirmed cases were defined as the presence of viral nucleic acid in blood specimens or by seroconversion or detection of increasing serum levels of specific IgM and IgG.

Second-line laboratory testing consisted of detection of DENV and CHIKV nucleic acids in plasma specimens by using real-time PCR and endpoint PCR, respectively, and detection of serum IgM and IgG by using an anti-CHIKV indirect immunofluorescence assay (Euroimmun AG, Lübeck, Germany), DENV IgG DxSelect (Focus Diagnostics, Cypress, CA, USA), and DENV IgM Capture DxSelect (Focus Diagnostics). Samples with DENV positive results by ELISA were further tested by plaque-reduction neutralization test to confirm specificity of antibody response.

A possible autochthonous case of WNF was defined as a patient with fever >38°C for <7 days, age >15 years, no recent travel history and absence of other obvious causes of fever.

A probable case was defined as a patient with a positive first-line laboratory test. The latter included detection of IgM and IgG antibodies against WNV in serum and CSF samples, with ELISA (WNV IgM capture DxSelect ELISA and IgG DxSelect ELISA kits, Focus Diagnostics, Cypress, California). A confirmed case was defined as a patient with at least one of the following laboratory criteria: isolation of WNV from blood; detection of WNV RNA by RT-PCR in blood; detection of increasing levels of IgM and IgG antibodies against WNV by ELISA, confirmed by plaque-reduction neutralisation test (PRNT).

### Entomologic surveillance

**Figure 4:**
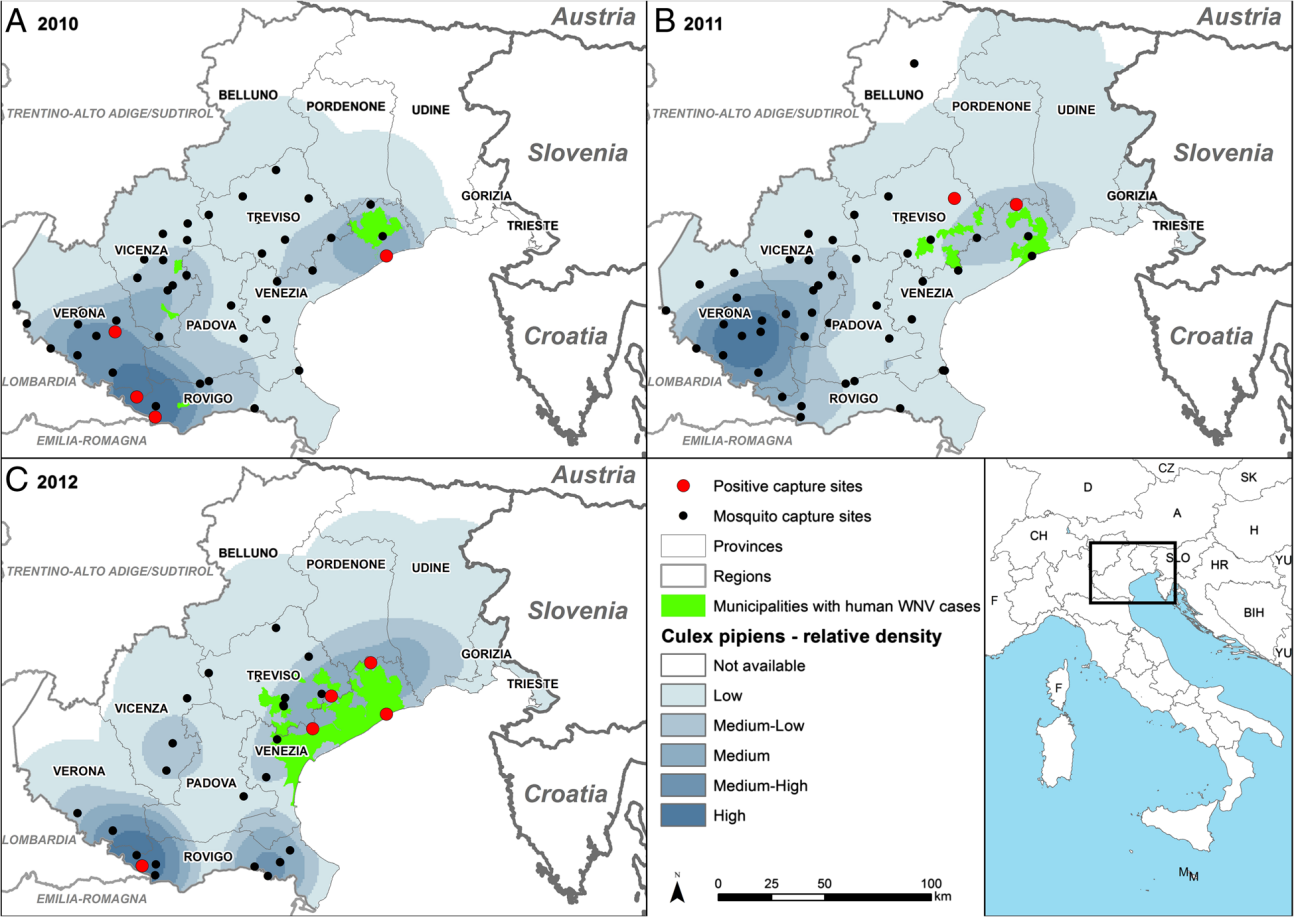
**Maps showing the relative density of *Cx. pipiens*, the mosquito traps activated and positive for WNV and the municipalities with human cases (WNF, WNND and donors) in 2010, 2011 and 2012.**

Entomologic surveillance for West Nile was carried out from May through November placing CDC-CO_2_ traps in five provinces of the region (Figure 4). Mosquitoes were collected fortnightly, identified, pooled in up to 50 specimens and tested using a One-Step SYBR Green-based Reverse Transcriptase-Real-Time PCR [16]. Amplicons were directly sequenced and positive samples confirmed by the National Reference Centre for Exotic Diseases (CESME, Istituto G. Caporale, Teramo).

Entomologic surveillance was also performed in areas surrounding the residence of human cases of DENV and CHIKV, if notified as viremic. In this case BG-sentinel traps with BG-Lure attractant (a mosquito attractant that mimics human skin odour) were used to capture adults of *Ae. albopictus*. Mosquitoes were counted, identified, frozen (-80°C) and sent to the National Arbovirus laboratory in Rome for virus examination.

### Statistical analysis

The mean abundance of mosquitoes per traps was compared among years using analysis of variance (ANOVA), after log_e_(x + 1) transformation of the data. The rates of infection in mosquitoes were adjusted for pooled samples calculating the estimated rate of infection (ERI), according to Cowling et al. [17]. For the 2012 season only, when a higher number of human cases was reported, the vector index was calculated for 2-week time steps by using abundance (numbers per trap per night) of the traps located in a radius of 25 km from the positive/s traps and ERI for *Cx. pipiens* mosquitoes [18]. For the calculation of the vector index a trap of the neighbouring region (Friuli Venezia Giulia) was also included due to landscape homogeneity and trap proximity with human cases. The vector index was correlated with the human cases using linear regression. The software used was SPSS for Windows, version 13.0.

### Ethical approval

This paper describes the results of a surveillance program put in place by health authorities of the Region and was not primarily intended as a research project. For this reason a formal ethical clearance was not required. Patient data are fully anonimized and no specific activity on human subjects was undertaken, other than that planned as regular surveillance activity and patient clinical management according to usual practice.

## Results

### Human surveillance

The main results are summarised in Table 1. In the 3 years of surveillance, between 2010 and 2012, 234 patients with fever after travelling were screened, of which 27 (11,5%) were found infected (24 with DENV and 3 with CHIKV). No autochtonous case was detected. Autochtonous patients screened for WNF were 408, and 24 (5,9%) were found infected. Some relevant details of the patients are reported in Table 2. Most patients with WNF (22/25, 88%) were hospitalized. No severe case of DENV (DHF, DSS) nor of CHIKV were observed. The median diagnostic delay since the onset of symptoms was 14 days for DENV (range 2-37), 17 days for CHIKV (range 16-19), 23 days for WNF (range 6-66).

**Table 2:** **Characteristic of DENV, CHIKV, WNF cases, diagnosed in Veneto Region between 15**^**th **^**of June and 30**^**th **^**of November, years 2010-2012**

	**N° of cases**	**Sex M/F**	**Mean age (range), years**	**Area of exposure**	**Time from symptom onset to diagnosis, (range) days**	**Positivity of PCR and/or viral isolation**	**Sero type**	**Adm. to hospital**
**DENV**	24	14/10	38 (17-64)	America (7)	14 (2-37)	15/24 (62%)	1 (5 cases)	n.a.
				Asia (15)			2 (3 cases)	
				Africa (1)			3 (5 cases)	
				Europe (1)				
**CHIKV**	3	1/2	29 (13-58)	Asia (3)	17 (16-19)	0/3 (0%)	-	n.a.
**WNF**	24	18/6	59 (29-80)	Italy (24)	23 (6-66)	4/24 (17%)	-	22/25 (88%)

### Entomologic surveillance

The number of traps activated in the three years and the results of entomologic surveillance for WN are summarized in Table 3. Seventeen species of mosquitoes were identified, and *Cx. pipiens* accounted for more than 80% of mosquitoes collected.

**Table 3:** **CO**_**2 **_**traps activated from 2010 to 2012 in Veneto region, mosquitoes collected, WNV positive pools and Estimated Rate of Infection (ERI)**

**Year**	**Total no. of traps**	**Total mosquitoes collected**	***Cx pipiens *** **(%)**	**mean *Cx pipiens*/capture**	**WNV pos pools**	**ERI min-max**	**WNV pos sites**
**2010**	43	137,848	86.9	249.9	10	0.056-2.174	4
**2011**	48	79,410	81.4	113.9	2	2.322-2.535	2
**2012**	24	104,930	74.7	291.3	11	0.052-0.946	5
***Total***	***115***	***322,188***	***81.6***	***199.9***	***23***	***0.052-2.535***	***9***

The WNV was found in 10, 2 and 11 pools of *Cx. pipiens* from 2010 to 2012 respectively, in sites of Rovigo, Verona, Venezia and Treviso provinces (Figure 4), areas where viral circulation was known, on the basis of human and veterinary cases detected in the previous years.

Despite the drought, the year 2012 was characterized by a significantly higher abundance of *Cx pipiens* compared to the two previous years (Table 3).

**Figure 5:**
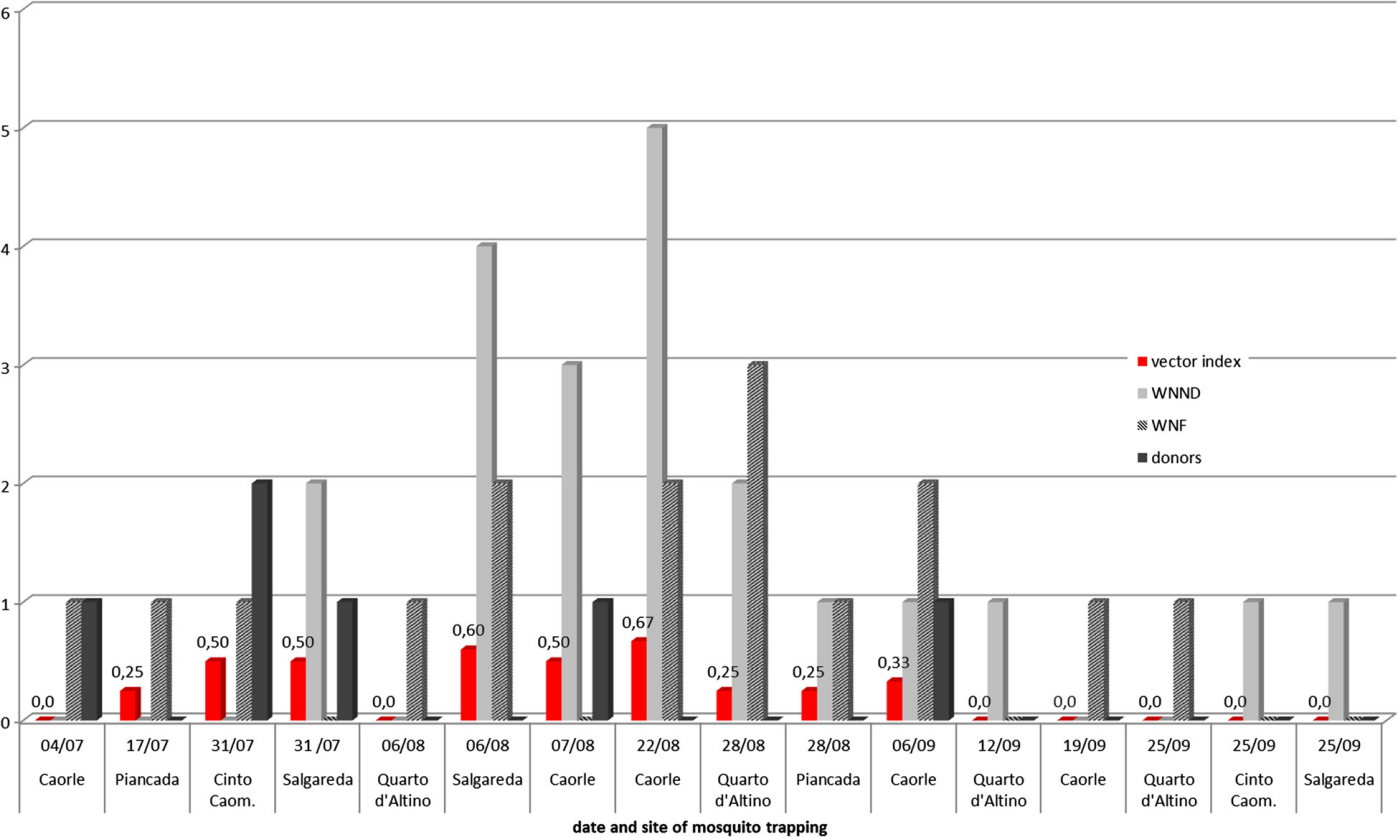
**Relationship between vector index and human cases reported in the 15 days following the record of a WNV positive trap in 2012.** The day of the onset of symptoms and the day of donation were considered for WNF/WNND and donors, respectively. When human cases are reported in the absence of mosquito positivity, a vector index = 0 in the closest trap in the previous two week has been reported.

The vector index ranged from 0.25 to 0.67 and was correlated with the increasing number of human cases reported in the following 15 days (R = 0.843, p < 0.01), including WNF, WNND and asymptomatic donors (Figure 5). In particular, single human cases were not or rarely predicted by the vector index, while clusters of cases (>2) were predicted by a vector index of ≥ 0.5 until the third week of August and by a vector index of ≥ 0.25 later in the season. Entomologic surveillance for *Ae.albopictus* was performed following three reports of dengue fever in Vicenza (June 26th) and Verona provinces (August 26th and September 7th) in 2011. Adults captured were 10, 22 and 7, respectively and all resulted negative for the virus. In 2012, two dengue viremic cases were notified (August 29^th^ and October 12^th^), however entomological surveillance was not required due to adverse weather conditions not compatible with *Ae. albopictus* presence in the respective sites.

## Discussion

In summary, during the three years period of the study we found 24 cases of DENV, 3 cases of CHIKV, 24 cases of WNF. All survived. The proportion over all subjects screened was 10.2% for DENV, 1.2% for CHIKV, 5.9% for WNF. While no *Aedes albopictus* adult was found infected with DENV or CHIKV, pools of *Culex pipiens* infected with WNV were found in 4 of the 7 provinces of the region.

The recent major outbreak of dengue in Madeira island (Portugal) [10], the previous local transmission of DENV documented in France [6,7], in Croatia [8,9] and the outbreak of CHIKV in Emilia Romagna in 2007 [5] highlight the importance of surveillance of imported cases in order to prevent or limit local transmission.

It is of note that PCR resulted positive in 15/24 (62%) of DENV cases in our series (Table 2): early detection in humans (that also allowed the identification of the serotype) prompted, when/where indicated, entomologic surveillance for *Ae. albopictus* to test the adults for viral presence, although none resulted positive.

Veneto is the only Italian region that has reported WNV human cases every year since 2008. In the whole country the data aren’t homogeneous [19,20]. The first human cases were reported in Veneto and in Emilia Romagna in 2008 [2,3], although a retrospective study revealed a human case in Tuscany in 2007 [21].

In 2011 two more regions, Sardinia and Friuli-Venezia Giulia, reported WNND cases [19] and one region, Marche, reported a WNF case [22]. If surveillance was limited to WNND, we would be completely unaware of the virus circulation in the latter region. Moreover the viral circulation had been documented to occur in many areas of Italy from North to South through retrospective screening of solid organ donors and through entomologic and veterinary surveillance [23].

Based on our findings, we suggest that surveillance should also include WNF in order to provide additional information on the viral circulation in humans and contribute to an early alert system.

Before the special surveillance, in two years time since the beginning of the outbreak of autochthonous WNV disease, only one retrospective case of WNF had been reported, in spite of the expected proportion WNF/WNND of 20/1, according to the literature [24]. It is worth noting that recent studies [25,26] suggest that the proportion of symptomatic patients is even higher than previously reported [25]. WNF is therefore the most common clinical presentation of West Nile disease, although many WNF cases remain undiagnosed. Patients with “simple” WNF should be monitored clinically to avoid the onset of complications, also remembering that WNF cases not evolving in WNND aren’t necessarily benign [27]. Moreover the epidemiology of WNV transmission is unpredictable. In United States in 2012, the largest outbreak ever recorded was observed, after years of relative quiescence. Petersen and Fischer consider WNV “unpredictable, disagreeable and difficult to control”, suggesting that “WNV will cause sporadic cases and big and small unforeseeable outbreaks for decades to come” [28]. For this reason they recommend a timely surveillance on a large scale in animals, insects and people.

In Veneto the entomological surveillance was useful (i) to define the mosquito species composition and relative density all over the region, indicating the area at major risk of WNV life cycle amplification, which are the main targets of the control strategies and (ii) to indicate *Cx. pipiens* as the main vector of WNV in this area.

The vector index showed to be correlated with the increasing number of human cases, indicating that it can be used as a predictor of clusters of cases in the surrounding areas within two weeks. However sporadic cases in humans (WNF and asymptomatic blood donors) have preceded detection in mosquitoes (Figure 5).

A clear vector index threshold was not identified and more data are needed before drawing any conclusion. In this respect, a more intensive entomological surveillance on a 1-week step has been scheduled for the next mosquito season in the area where the majority of human cases have been reported.

Interestingly, the 2012 upsurge of WNV human cases was found in a year characterized by a strong drought, which did not cause a decrease of mosquito population, likely due to the artificial irrigation in this area strongly devoted to corn production. The concentration of larval breeding sites closer to human settlement caused by the irrigation and the contemporary presence of synantropic birds able to act as reservoir of WNV may have caused an increased contact between humans and infected mosquitoes.

The major strengths of this surveillance project are the good integration of human and entomologic surveillance and the ability to provide a sufficiently comprehensive picture of the occurrence of these three arboviruses of human interest in our region.

The project had some limitations, too. A major weakness is the low number of WNF cases compared to WNND. The main reason is that most mild cases do not seek medical advice and, if they do, are seldom referred to hospital or to a laboratory. The cases detected are therefore the tip of the iceberg and, not surprisingly, were almost all admitted. Also, the total number of febrile patients returning from endemic countries and screened for DENV and CHIKV is comparatively low. Moreover, as most of them come from countries where malaria also occurs, it is clear that all these patients should promptly referred to skilled diagnosis, which is not always the case. In order to deal with both weaknesses, a better involvement of family doctors in surveillance is clearly crucial.

Future research should focus on the sustainability of this surveillance program, including a thorough cost analysis, in order to be able to recommend its adoption as a routine surveillance system and its diffusion to other regions.

## Conclusions

WNV is likely to cause sporadic cases and unforeseeable outbreaks for decades. Including WNF in regular surveillance provides a more comprehensive picture of WNV circulation in humans. Timely detection of DENV and CHIKV should prompt vector control measures and contribute to prevent local outbreaks.

## Competing interests

All authors declare that they do not have any competing interest.

## Authors’ contributions

FG and ZB conceived the study design, wrote the study protocol, concurred to data analysis, wrote the draft and final version of the manuscript. GC conceived the study design, supervised the entomologic surveillance and wrote the final version of the manuscript (entomologic data). FM carried out the entomologic surveillance of *Cx. Pipiens.* PM elaborated the maps and statistical analysis (entomologic data). AD carried out the entomologic surveillance of *Ae. Albopictus.* GN and FR concurred to the study design and to the study protocol. FZ entered data in database, concurred to data analysis. GP and LB coordinated the laboratory activities. ER, AMC, GC, PR, MG, AA, MF diagnosed and managed cases. All authors read and approved the final version of the manuscript.

## Pre-publication history

The pre-publication history for this paper can be accessed here:

http://www.biomedcentral.com/1471-2334/14/60/prepub
